# A Comprehensive Molecular Interaction Map for Rheumatoid Arthritis

**DOI:** 10.1371/journal.pone.0010137

**Published:** 2010-04-16

**Authors:** Gang Wu, Lisha Zhu, Jennifer E. Dent, Christine Nardini

**Affiliations:** Group of Clinical Genomic Networks, CAS-MPG Partner Institute for Computational Biology, Shanghai Institutes for Biological Sciences, Chinese Academy of Sciences, Shanghai, People's Republic of China; University of Western Ontario, Canada

## Abstract

**Background:**

Computational biology contributes to a variety of areas related to life sciences and, due to the growing impact of translational medicine - the scientific approach to medicine in tight relation with basic science -, it is becoming an important player in clinical-related areas. In this study, we use computation methods in order to improve our understanding of the complex interactions that occur between molecules related to Rheumatoid Arthritis (RA).

**Methodology:**

Due to the complexity of the disease and the numerous molecular players involved, we devised a method to construct a systemic network of interactions of the processes ongoing in patients affected by RA. The network is based on high-throughput data, refined semi-automatically with carefully curated literature-based information. This global network has then been topologically analysed, as a whole and tissue-specifically, in order to translate the experimental molecular connections into topological motifs meaningful in the identification of tissue-specific markers and targets in the diagnosis, and possibly in the therapy, of RA.

**Significance:**

We find that some nodes in the network that prove to be topologically important, in particular AKT2, IL6, MAPK1 and TP53, are also known to be associated with drugs used for the treatment of RA. Importantly, based on topological consideration, we are also able to suggest CRKL as a novel potentially relevant molecule for the diagnosis or treatment of RA. This type of finding proves the potential of *in silico* analyses able to produce highly refined hypotheses, based on vast experimental data, to be tested further and more efficiently. As research on RA is ongoing, the present map is *in fieri*, despite being -at the moment- a reflection of the state of the art. For this reason we make the network freely available in the standardised and easily exportable .xml *CellDesigner* format at ‘www.picb.ac.cn/ClinicalGenomicNTW/temp.html’ and ‘www.celldesigner.org’.

## Introduction

Rheumatoid Arthritis (RA) is a complex disease involving a yet unknown number of genes, and affecting a large number of organs, tissues and sites across the body. It is affecting approximately 1% of the population worldwide [1], with this rate rising for the first time in 40 years, as reported at the American College of Rheumatology meeting in San Francisco (CA, USA) in 2008. RA is a systemic autoimmune disease causing recruitment and activation of inflammatory cells, synovial hyperplasia, and destruction of cartilage and bone. A complete loss of mobility and functioning can be the final evolution of the disease [2]. Although RA involves the synovial joints, it presents several systemic features as, in fact, several other organs are affected including skin, lungs, kidneys, blood vessels and heart [3]–[6]. Because of its complexity, having a broad, systemic perspective on the biological functions activated and the molecular pathways involved in the disease is of crucial importance.

In this direction several types of approaches and data platforms can be used for investigation. Genome-Wide Association studies (GWAs) scan the whole genome in search of loci susceptible to carry mutations related to RA (only as a sample of very recent studies [7]–[9]). Gene microarray data have contributed greatly to pathogenesis and to the identification of biomarkers for diagnosis, to patient stratification and prognostication of RA [10]. Other studies join the information from these 2 approaches and compare differentially expressed genes with genome-wide association studies to better predict candidate susceptibility genes of RA [11]. Furthermore, some signal transduction pathways have also been identified as being involved in the disease progression and in the effects of therapies of RA. The TGF-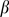 pathway, for example, shows broad, constitutive alternation in Rheumatoid Arthritis Synovial Fibroblasts (RASFs) [12] and the NF-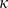
B pathway has been inhibited during the anti-TNF-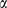 therapy by etanercept [13]. The signal transduction pathways in RA and some of the important proteins of these pathways have been identified as drug targets to treat RA [14]–[16]. However, due to the complex interactions of these pathways, treatments that target only one protein may not be very effective. Besides the relevance of proteins as targets, a recent study has also shown that miR-155 was up-regulated during the treatment with TNF-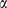 in RASFs [17]. This implies that some microRNA may be involved in RA progression. Due to the complexity of RA, however, the interaction among all of these molecules and pathways is still obscure. This is highly relevant in the identification of new therapies, as in fact, some of the most common drugs used to treat RA, such as MTX (Methotrexate), can cause liver, lung and kidney damage, as well as strong immunodepression. To avoid these important side effects and to develop more specific and useful drugs, the whole structure of the molecular networks involved in RA needs to be studied and clarified. The identification and analysis of this complex map cannot be performed without the help of computational biology. Hence we present here a comprehensive map for RA that combines together the molecules and pathways that were so far found to be associated with RA, based on systemic, high-throughput data, and made available following the format suggested by *CellDesigner* [18], a popular and successful standard for the exchange of cellular maps [19].

**Figure 1:**
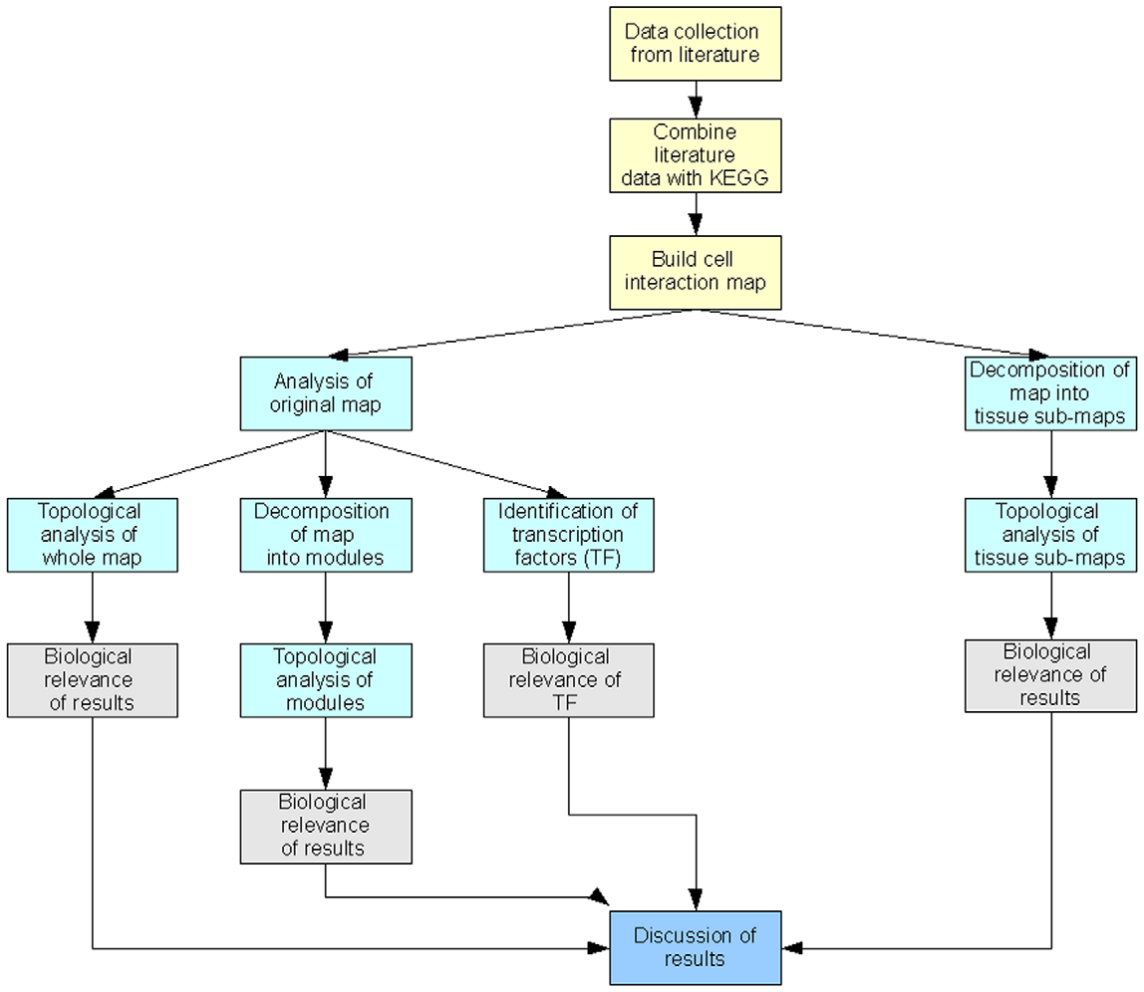
Framework of building and analysis of molecular interaction map. Pale-yellow = data collection and map building (materials and methods), pale green = map analysis (results), grey = biological interpretation (results), blue = discussion.

To date, the most abundant source of high-throughput, systemic, genome-wide data is still represented by microarrays for gene expression, although soon this may be replaced by more quantitative information from mRNAs sequencing [20], [21]. For this reason, in order to build a comprehensive map of the processes ongoing in RA, we chose to construct a molecular map based on the results of high-throughput analyses. In fact, despite the growing availability of proteomic data and their promising applications, the throughput remains lower and limited to a number of validated targets [22]. In order to give a systemic description of the relationships among the genes and pathways known to be involved in RA, we merged the information of all available papers related to high-throughput experiments (mRNA, miRNA) on RA ( [11]–[13], [17], [23]–[46]). Using these information and further data available from the Kyoto Encyclopedia of Genes and Genomes (KEGG) ‘http://www.genome.jp/kegg/’, we build a comprehensive cell-level interaction map. Visually, we present this molecular-interaction map as a gene regulation map and a protein-protein interaction map, linked by a number of transcription factors. We then use network analysis methods to analyse the map as a whole and tissue-specifically. In Figure 1 we present a schematic view of the study framework.

Basically, our map uses the information retrieved from the results of functional genomic analyses on RA (in the form of differentially expressed genes), as a blueprint for the construction of a more detailed interaction map based on assessed literature (in the form of pathways). It is very clear that such a map is likely to include a number of false positives, since the differential expression of genes may not correspond to the effective presence of the corresponding protein. However, the additional layer of information retrieved from pathways stored in literature shows that the reconstructed map is able to identify hubs that are known targets of current RA treatments, and to suggest interesting new targets. Moreover, one of the preeminent characteristics of *in silico* modeling and analysis is to offer ‘cheap’ working hypotheses to be subsequently tested *in vitro* and *in vivo*. In this respect our analysis highlights the importance of proteins that have already been identified as relevant in the evolution of RA, giving more insight into their potential role, and in particular into their ability to affect neighboring pathways and functions. This type of analysis indicates potential new markers in the diagnosis and/or monitoring of RA, that is markers that could be loaded, for example, on board of point-of-care diagnostic tools, based not only on genomic (DNA) screens [47], but also on functional genomic (mRNA) screens [48]. Generally, the mRNAs on which we base our analysis are identified as relevant under different biological conditions, such as healthy versus diseased subjects, RA versus other immune diseases, or comparing subjects before and after treatment. The experiment samples are also from different tissues, such as peripheral blood mononuclear cell (PBMC), synovial fibroblasts and cartilage.

A range of network analysis methods have been successfully applied in multiple studies in an attempt to understand the structure of interaction networks, or the effect that single genes or molecules have on such networks (see [49]–[53] for example). In the ‘Analysis of Molecular-Interaction Map and Network Modules’ section of this paper we use network analysis methods to understand the systemic interactions of molecules involved in RA. We begin by determining the topological parameters of the network and analysing the structure of the map as a whole. Many biological networks display scale-free properties [54]–[58], which means that they contain fewer nodes that have many connections to other nodes' hubs. Targeting hubs enables one to reach several other nodes in a shorter time frame than would be possible by targeting nodes at random. On the downside, the presence of hubs in scale-free networks means that the network can be more easily destroyed if the hubs are removed. We use the degree distribution, which describes the number of links per node, to identify hubs and to determine if the network is scale free (if it is, then the degree distribution follows a power-law distribution). We then consider whether the hubs in the molecular-interaction map have known biological relevance. Further to understanding the structure of the network through the presence of hubs, we decompose the network into modules according to its structure. We expect the network to have a bow tie structure [56], which means that it can be separated into four components: a central part containing strongly connected components, an IN component containing nodes from which the central component can be reached, an OUT component containing nodes that can be reached from the central component and a fourth component containing all other nodes. Within the central component of the bow tie structure, we can identify topologically relevant cycles of nodes. A cycle in this sense is a group of nodes that are connected to each other such that the links between nodes form a cycle containing all nodes. Definition of cycles in a cell can represent biologically significant features, such as feedback in the cell, which is an important way for the cell to regulate different biological mechanisms, such as protein-protein interactions, gene-regulation or metabolic pathways [59], [60]. We use the relevant cycles and consider the paths attached to them, thus creating separate modules from the interaction-network whose core components are a closed cycle. We then look for biological relevance in these newly defined modules. We determine if the modules produced show similarities to biological modules (in the sense that they may act as an independent sub-system or perform a specific biological function in the cell) [50]. This module analysis helps one to decompose the complex network and furthermore identify the pathways involved in RA. By careful dissection of the pathways, novel therapeutic interventions designed to block signaling may be developed. Several potential targets, including MAP kinases and NF-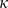
B, are already being explored. Analysis of the interaction network without any amount of decomposition will not give a full understanding of its structure, which is important for thorough biological interpretation.

Given that transcription factors have been shown to be potential drug-targets, and that it has also been shown that it is possible to modulate some transcription factors through signaling cascades, our attention is drawn the transcription factors in our network. In ‘The Role of Transcription Factors’ we investigate whether the transcription factors present in our network also have important topological properties, in the sense that they link topologically distinct parts (i.e. different modules) of the network. If this is the case, then it may be possible to influence the different topologically important parts of the cell, by concentrating on specific transcription factors.

Further to analysis of the interaction map as a whole, in section ‘Analysis of Tissue-Specific Networks’ we also present the results of a tissue-specific analysis. Here we consider whether there are topological and biological differences in the way in which various tissue types act within the cell with respect to RA. By assigning a species tag to each node in the molecular-interaction map, we produce five tissue-specific sub-maps (Blood Peripheral Blood Mononuclear Cell (Blood_PBMC), Blood Peripheral Blood Mononuclear Cell plus Polymorphonuclear leukocytes (Blood_PBMC_PMN), cartilage, Synovial Fibroblast and synovial Polymorphonuclear leukocytes (synovial_PMN)). Of these five sub-maps, we are only able to achieve meaningful topological results for three (Blood_PBMC, Synovial Fibroblast and cartilage), due to the small amount of data used to build the remaining two sub-maps. For these three larger sub-maps, we pay particular attention to the identification of hubs by tissue type, and to areas where there is an overlap between tissue types. The results from this part of the study enable us to comment on whether there exist tissue specific markers that could play a role in the diagnosis of RA.

Throughout the analysis, we constantly are required to return to the literature in order to determine if the topological results have any biological significance. We present our findings and identify areas for further research.

## Results

The results section can be broken down as shown in Figure 1. In the first, and largest, section we present the results from the analysis of the molecular-interaction map as a whole and broken down into topologically significant modules as previously discussed. In this first section, we determine the biological relevance of hubs in the molecular-interaction map as well as in the three largest modules. We find, on the whole (and particularly for the molecular-interaction map when it is analysed before any sort of decomposition), that the hubs in the molecular-interaction map are already known drug targets. This is consistent with the role of a hub in a network and is therefore of no surprise. Interestingly, however, only one of the hubs in the molecular-interaction map is a transcription factor, implying that the transcription factors that act as a bridge between the gene-regulatory and the protein-protein interaction maps, do not directly link a large number of nodes. The role of hubs, as well as explanation for the biological significance of each of the modules, is then presented. Next, we consider the transcription factors present in the molecular-interaction map and discuss the role of these transcription factors from a biological point of view. Finally, we present the results from the tissue-specific analysis.

**Figure 2:**
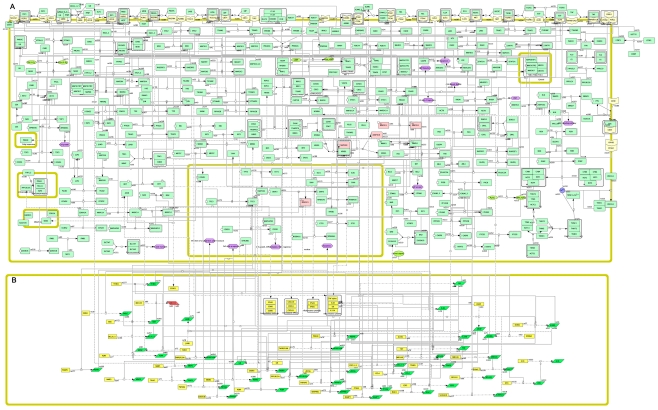
Molecular-interaction map for Rheumatoid Arthritis. A = protein-protein interaction map, B = gene regulation map. The two maps are joined by transcription factors.

Figure 2 shows the molecular-interaction map as displayed in *CellDesigner 4.0.1*. The map is separated into a protein-protein interaction (A) and a gene regulation map (B). The two maps are linked by transcription factors identified from the molecular-interaction map (discussed below). The resulting map has a total of 273 proteins, which are represented in 348 distinct chemical species (248 of them are located in the cytoplasm, 44 in the membrane, 21 in the nucleus, 25 in the outside of the membrane, 4 in the mitochondrion, 1 in the cytosol, 4 in the endoplasm reticulum, 1 in the golgi apparatus) and 255 reactions and regulations (among them, 24 protein associations, 3 protein dissociations, 160 state transitions, 47 transcriptional regulations, 10 protein translations, 7 transportations, 2 *known_transition_omitted*, indicating an indirect interaction, and 2 *unknown_transitions*, indicating interactions predicted but not validated from literature [24]). The genes associated with RA distribute almost every organelle of the cell including Golgi apparatus, endoplasmic reticulum and mitochondrion.

### Analysis of Molecular-Interaction Map and Network Modules

#### Molecular-Interaction Map

**Figure 3:**
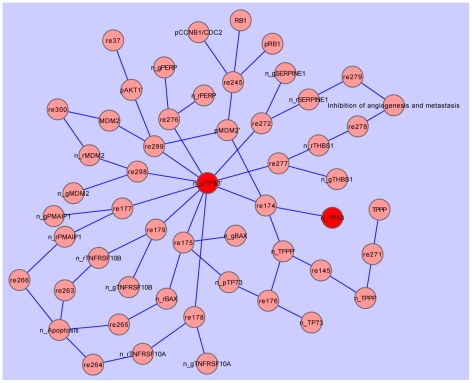
*Cytoscape* view of Module 10, with TP53 hub highlighted (red). n = nucleus, p = phosphorylation, g = gene, r = RNA, ’ = active.

The systemic network of interactions was first analysed as a whole and then decomposed in to topologically significant modules. The map and all modules are available for public use at ‘http://www.picb.ac.cn/ClinicalGenomicNTW/temp.html’. The original network, which has a total of 776 nodes and 886 edges, displays scale-free properties, as expected from a biological network [56], with the in- and out-degree distributions having power law exponents of approximately 2.394 and 2.479 (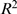 value of 0.951 and 0.948) respectively. The network consists of 23 connected components, with each node having an average of 2.281 neighbours (network density 0.003). We can therefore conclude that the network is not well-connected. Analysis of shortest path frequency shows that the mean shortest path length is approximately 16.042 (variance = 75.80) and network diameter (maximum length of shortest path) of 48. Using node degree to identify network hubs, we identify six protein hubs (AKT2, EGFR, IL6, MAPK1, RAC1,2 and TP53). An example of a protein hub (TP53), and its connections, is shown in Figure 3.

#### Biological Relevance of Hubs in the Molecular-Interaction Map

We describe in this section the biological properties of the hubs in the whole map. Interestingly, they are all related to RA therapy.

AKT2 is a putative oncogene encoding a protein belonging to a subfamily of serine/threonine kinases, containing SH2-like domains. Itis related to various kinds of antitumor drugs such as cetuximab, erlotinib, gefitinib and lapatinib. All of these drugs are associated with the EGFR inhibitor pathway, which plays an essential role in regulating cell division and death [61]. AKT2 is the hub of Module 4 and belongs to 20 different pathways (except hsa04912, 04650, 04810, 04540, 04320 and 04670, see below). AKT2 is also one of the synovial genomic targets of bucillamine, mostly used as treatment in Japan to reduce pain in RA patients [45].

EGFR is a transmembrane glycoprotein that is a member of the protein kinase superfamily. This protein is a receptor for members of the epidermal growth factor family. EGFR is a cell surface protein that binds to epidermal growth factor. Binding of the protein to a ligand induces receptor dimerization and tyrosine autophosphorylation and leads to cell proliferation. It is associated with some drugs to treat neoplasms such as canertinib [62], cetuximab [63] and cisplatin [64], [65].

IL6 (interleukin 6) encodes a cytokine that functions in inflammation and the maturation of B cells. The protein is primarily produced at sites of acute and chronic inflammation, where it is secreted into the serum and induces a transcriptional inflammatory response through IL6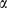 receptor. The functioning of this gene is implicated in a wide variety of inflammation-associated disease states, including susceptibility to diabetes mellitus and systemic juvenile RA. It has been found that the functional dynamics of IL6 might be crucial for the outcome of etanercept therapy and the particular role of IL6 in RA is presently considered in therapeutic interventions that target IL6 or its receptor [13]. The expression of IL6 is also reduced in RASFs treated with trichostatin A and ultrasound [26]. IL6 was also inhibited by SB-203580, a p38 MAPK inhibitor, in IL1B treated broblast-like synoviocytes (FLS) cells [31]. IL6 had also been reported to be associated with immunosuppressive effects of glucocorticoids. Dexamethasone was found to be a potent inhibitor of IL6 gene in connective tissue-type cells from the synovium of patients with RA [66]. This was mainly because dexamethasone can decrease the mRNA stability of IL6 gene [67].

MAPK1 is a member of the MAP kinase family. MAP kinases act as an integration point for multiple biochemical signals, and are involved in a wide variety of cellular processes such as proliferation, differentiation, transcription regulation and development. The activation of this kinase requires its phosphorylation by upstream kinases. Upon activation, this kinase trans-locates to the nucleus of the stimulated cells, where it phosphorylates nuclear targets. It is also related to some immune regulative drugs, such as cetuximab, and drugs used to treat cancers, such as isoproterenol [68], erlotinib and sorafenib [69].

RAC1 and RAC2 are GTPases which belongs to the RAS superfamily of small GTP-binding proteins. Members of this superfamily appear to regulate a diverse array of cellular events, including the control of cell growth, cytoskeletal reorganization, and the activation of protein kinases. It has been shown that Azathioprine-generated 6-Thio-GTP can prevent the development of an effective immune response via blockade of Vav activity on RAC proteins [70]. Azathioprine is also a drug used to treat RA [71].

TP53 responds to diverse cellular stresses to regulate target genes that induce cell cycle arrest, apoptosis, senescence, DNA repair, or changes in metabolism. It is a DNA-binding protein containing transcription activation, DNA-binding, and oligomerization domains. TP53 is postulated to bind to a p53-binding site and activate expression of downstream genes that inhibit growth and/or invasion, and thus function as a tumor suppressor. Methotrexate is an antimetabolite and antifolate drug used in treatment of cancer and autoimmune diseases. It acts by inhibiting the metabolism of folic acid. *In vitro* studies have shown that methotrexate caused single- and double-strand DNA breaks is associated with TP53 [72]–[74]. The p53 pathway was also found to be affected by bucillamine, which, as said above, is mainly used as a treatment in Japan to reduce pain in RA patients [45].

#### Network Modules

By considering relevant cycles in the interaction network (see Materials and Methods), we were able to decompose the interaction network into 11 network modules whose core components were represented by such cycles within the interaction network. A final 12th module was created from weakly connected components that were disjoint from all other modules. Table 1 gives the results from a topological analysis of all modules. Hubs were identified in the three largest modules (Modules 1,2 and 4) as discussed below.

**Table 1:** Topological Analysis of Modules and Tissue Maps.

Network	Node	Edge	Comp	Nei	Path	Dia	Den	Din	Dout	Rin	Rout
Main	776	886	23	2.28	16.04	48	0.003	2.39	2.48	0.95	0.95
Mod 1	111	120	1	2.13	10.71	26	0.019	1.6	2.73	0.84	0.86
Mod 2	173	182	1	2.10	15.67	39	0.012	2.269	3.043	0.96	0.99
Mod 3	75	85	1	2.27	5.83	14	0.031	1.714	2.389	0.85	0.92
Mod 4	72	82	1	2.28	5.85	14	0.032	1.664	2.286	0.85	0.81
Mod 5	12	16	1	2.67	3.33	7	0.242	1.063	1.661	0.87	0.75
Mod 6	43	48	1	2.23	3.08	8	0.053	1.664	2.635	0.89	0.95
Mod 7	11	11	1	2	3.32	7	0.2	1.807	1.807	1	1
Mod 8	4	4	1	2	2	3	0.667	na	na	na	na
Mod 9	20	23	1	2.3	2.40	6	0.121	1.07	1.594	0.84	0.88
Mod 10	53	59	1	2.23	4.71	12	0.043	2.175	1.576	0.93	0.88
Mod 11	6	6	1	2	2.2	4	0.4	na	na	na	na
Mod 12	74	49	25	1.32	1.76	4	0.018	2.605	4.492	0.78	1
B_PBMC	450	428	65	1.90	6.09	20	0.004	2.279	2.964	0.97	0.91
B+PMN	3	2	1	1.33	1.33	2	0.667	na	na	na	na
Cart	50	30	21	1.2	2.37	6	0.024	2.634	3.7	0.987	1
SF	301	236	75	1.57	3.98	12	0.005	2.951	4.129	0.97	0.95
S_PMN	16	10	6	1.25	2.36	6	0.083	1.585	na	1	na

Node = Number nodes, Edges = Number edges, Comp = Number of connected components, Nei = Average number of neighbours, Path = Average shortest path, Dia = network diameter, Den = Network Density, Din = In-degree distribution power law exponent, Dout = Out-degree distribution power law exponent, Rin = 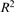 value for in-degree distribution power-law fit, Rout = 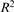 value for out-degree distribution power-law fit, Main = cell interaction map, Mod = Module, B_PBMC = Blood_PBMC, B+PMN = Blood_PBMC plus PMN, Cart = cartilage, SF = Synovial Fibroblast, S_PMN = Synovial_PMN.

Module 4 is the largest module with 292 nodes and 334 edges. It has a density of 0.008 and in- and out-degree distributions that can be fitted to power-law distributions with exponent values of 2.009 and 2.352 respectively (corresponding 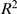 values 0.961 and 0.939). Module 4 has six protein hubs: AKT2, MAPK1, EGFR, CRKL, GNAI3 and FGFR1. Module 2 is the next largest module, with 173 nodes and 182 edges. The density of Module 2 is 0.012 and its in- and out-degree distributions also have a good fit to the power-law distribution (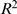 values of 0.96 and 0.989), with exponent values of 2.269 and 3.043 for in- and out-degree respectively. Module 2 has two protein hubs, namely MAPK14 and IL6. The third largest module, Module 1, has 111 nodes and 120 edges. The density of this module is 0.019. Due to the smaller number of nodes with high degree, the fit of a power-law to the degree distributions for Module 1 is less accurate, with 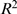 values dropping to 0.843 and 0.862 for in- and out-degree distribution. Module 1 has only two protein hubs, MAPK8 and the complex containing RAC1 and RAC2. As the size of other modules quickly decreases, with approximately half of the modules containing fewer than 50 nodes, we do not discuss their topological features here.

#### Biological Relevance of Hubs in Modules

Module hubs that are also hubs in the molecular-interaction map (namely AKI1, MAPK1, EGFR, IL6 and RAC1, RAC2) are not re-discussed in this section.

FGFR1 (Module 4) is a membrane protein. It is a member of the fibroblast growth factor receptor (FGFR) family, where amino acid sequence is highly conserved between members and throughout evolution. The extracellular portion of the protein interacts with fibroblast growth factors, setting in motion a cascade of downstream signals, ultimately influencing mitogenesis and differentiation. This protein is associated with palifermin, which is human keratinocyte growth factor used to treat mucositis. GNAI3 (Module 4) is associated with some antidepressants such as citalopram, fluoxetine, paroxetine and sertraline.

MAPK14 (Module 2) is also a member of the MAP kinase family, already described above. This kinase is activated by various environmental stresses and proinflammatory cytokines. The activation requires its phosphorylation by MAP kinase kinases (MKKs), or its autophosphorylation triggered by the interaction of MAP3K7IP1/TAB1 protein with this kinase. The substrates of this kinase include transcription regulator ATF2, MEF2C, and MAX, cell cycle regulator CDC25B, and tumor suppressor p53, which suggest the roles of this kinase in stress related transcription and cell cycle regulation, as well as in genotoxic stress response. Since this protein is one of most important members involved in the RAF/MEK/ERK signaling pathway, MAPK14 is associated with sorafenib, which is a drug used for the treatment of advanced renal cell carcinoma (primary kidney cancer) and advanced hepatocellular carcinoma (primary liver cancer). Sorafenib is a small molecular inhibitor of Raf kinase, PDGF (platelet-derived growth factor), VEGF receptor 2 & 3 kinases and c-Kit, the receptor for stem cell factor. A growing number of drugs target most of these pathways. The originality of sorafenib lays in its simultaneous targeting of the Raf/Mek/Erk pathway [69].

MAPK8 (Module 1) is a member of the MAP kinase family (see above). This kinase is activated by various cell stimuli, and targets specific transcription factors, and thus mediates immediate-early gene expression in response to cell stimuli. The activation of this kinase by tumor-necrosis factor alpha (TNF-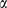) is found to be required for TNF-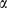 induced apoptosis. This kinase is also involved in UV-radiation induced apoptosis, which is thought to be related to cytochrome c-mediated cell death pathway. This protein is related to many antineoplastic drugs such as cetuximab, erlotinib and sorafenib [69]. This protein is also a potential target for therapeutic treatment of metabolic syndrome. MAPK8 activation in adipose tissue can cause insulin resistance in the liver [75].

#### Module Analysis

Using DAVID [76] (see Materials and Methods) and adopting both false discovery rate (FDR) and Bonferroni (FWER) correction to control the number of false positives, we were able to perform a pathway analysis on proteins involved in Modules 1, 2, 3, 4, 6 and 10. A table giving the full set of results for Modules 1, 2, 3, 6 and 10 is available in the Table S1 (Module 4 is discussed separately below - see also Table 2).

**Table 2:** Module 4 Pathway.

Pathway	Count	List Total	Bonferroni	FDR
hsa04010:MAPK signaling pathway	43	104	5.05E-23	3.15E-22
hsa04510:Focal adhesion	36	104	9.43E-20	5.88E-19
hsa04012:ErbB signaling pathway	23	104	2.40E-15	1.50E-14
hsa05220:Chronic myeloid leukemia	21	104	4.46E-14	2.78E-13
hsa05215:Prostate cancer	22	104	6.69E-14	4.22E-13
hsa04664:Fc epsilon RI signaling pathway	20	104	6.25E-13	3.90E-12
hsa05210:Colorectal cancer	21	104	6.92E-13	4.32E-12
hsa05211:Renal cell carcinoma	19	104	1.52E-12	9.47E-12
hsa05213:Endometrial cancer	16	104	5.26E-11	3.28E-10
hsa05212:Pancreatic cancer	18	104	1.20E-10	7.48E-10
hsa04620:Toll-like receptor signaling pathway	20	104	3.40E-10	2.12E-09
hsa04912:GnRH signaling pathway	19	104	8.54E-10	5.33E-09
hsa05214:Glioma	15	104	1.53E-08	9.53E-08
hsa04650:Natural killer cell mediated cytotoxicity	20	104	2.58E-08	1.61E-07
hsa05223:Non-small cell lung cancer	14	104	2.99E-08	1.87E-07
hsa04910:Insulin signaling pathway	20	104	5.09E-08	3.17E-07
hsa05218:Melanoma	15	104	1.14E-07	7.09E-07
hsa04810:Regulation of actin cytoskeleton	24	104	1.57E-07	9.81E-07
hsa04370:VEGF signaling pathway	15	104	1.70E-07	1.06E-06
hsa05221:Acute myeloid leukemia	13	104	8.77E-07	5.47E-06
hsa04660:T cell receptor signaling pathway	16	104	9.01E-07	5.62E-06
hsa04540:Gap junction	16	104	1.05E-06	6.54E-06
hsa04662:B cell receptor signaling pathway	12	104	0.00007	0.0004
hsa04320:Dorso-ventral axis formation	8	104	0.0007	0.004
hsa04670:Leukocyte transendothelial migration	14	104	0.0008	0.005
hsa04210:Apoptosis	12	104	0.0009	0.006

hsa = homo sapiens, FDR = false discovery rate.

Module 1 contains 56 proteins, of which we obtained information about 50 proteins (the other 6 proteins were not enrolled during the process of this DAVID pathway analysis). Among these 50 proteins, 17 proteins are involved in the Toll-like receptor signaling pathway, 14 proteins belong to Leukocyte transendothelial migration and 18 proteins come from the MAPK signaling pathway. Module 2 contains 80 proteins. Sixty-four of which were selected in the pathway analysis. Among these 64 proteins, 45 proteins are involved in the Toll-like receptor signaling pathway, 27 proteins belong to the MAPK signaling pathway, 16 proteins are associated with apoptosis and 16 proteins come from cytokine-cytokine receptor interaction. In Module 3, 23 proteins out of 30 proteins have been enrolled in the pathway analysis. According to the statistical significance, 8 proteins are related to the Toll-like receptor signaling pathway. Module 4 is the biggest module, containing 112 proteins. The DAVID pathway analysis of Module 4 showed 104 proteins distributed among 26 different pathways (see Table 2). In Module 4 there are 43 proteins from the MAPK signaling pathway, 36 proteins from focal adhesion, 23 proteins from the ErbB signaling pathway, and some cancer associated pathways such as leukemia, prostate cancer and colorectal cancer (Table 2). There are 23 proteins in Module 6, 22 proteins have been selected by the DAVID pathway analysis. Of these, 13 proteins are involved in the natural killer cell mediated cytotoxicity, 11 proteins are related to the T-cell receptor signaling pathway and 10 proteins belong to focal adhesion. In Module 10, the p53 signaling pathway has been highlighted by the DAVID pathway analysis, in which 11 proteins have been enrolled.

Most of these pathways have been found to be playing important roles in RA. For example, the Toll-like receptor signaling pathway is shared by Modules 1, 2, 3 and 4. The Toll-like receptors (TLRs) signaling pathways are membrane-bound receptors which are expressed in innate immune cells, such as macrophages and dendritic cells. TLRs signaling plays an important role in the activation and direction of the adaptive immune system by the up-regulation of co-stimulatory molecules of antigen presenting cells. The activation of the TLRs signaling pathway can trigger the activation of the MAPK and NF-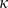
B pathways. Evidence is emerging that certain TLRs play a role in the pathogenesis of infectious and/or infammatory diseases. For example TLR4, which has also been mentioned in the tissue map analysis, is one of the most studied members of TLRs. TLR4 is a possible receptor for endogenous factors released during tissue injury and infammation, such as hsp60 [77]. One study has shown that TLR2 and TLR4 are expressed by RA synovial membrane cells and are able to up-regulate inflammatory cytokine production, which promotes the inflammatory and destructive process in RA [78]. There is considerable evidence from rodent models that activation of the TLRs can induce or exacerbate inflammatory arthritis, and TLR2 deficient animals exhibited a significantly reduced severity of arthritis [79].

The MAPK signaling pathway has been found to be involved in Modules 1, 2 and 4. Some of the members of MAPK signaling pathway have been identified as hubs in the topological analysis. MAPK is a key signal transduction pathway of inflammation, which shuttles information about inflammatory stimuli to the cell nucleus. All the MAPK signaling pathways in these 3 modules are triggered by IL1B, which is an important mediator of the inflammatory response and is involved in a variety of cellular activities, including cell proliferation, differentiation, and apoptosis. IL1B is also a hub in the cartilage tissue map (see below). The MAPK pathway plays an important in the development and progress of RA. For example, cartilage damage is a hallmark of RA. It is based on increased proteoglycan loss as well as attachment and invasion of inflammatory tissue into the cartilage, which leads to its structural disintegration. Production of matrix metalloproteinases (MMPs) by synovial tissue appears to be a key prerequisite for synovial tissue to invade and destroy cartilage. Synthesis of MMPs is regulated through multiple MAPK families, suggesting that a blockade of MAPK might have structural benefit in arthritis [80], [81]. Activation of stress kinase pathways ERK, JNK, and p38 MAPK is a typical feature of chronic synovitis during RA, and several proinflammatory mediators use the signaling of these stress kinase pathways [82].

The other pathways are connected to immunity and inflammation, focal adhesion, apoptosis and cancers. Most of the hubs mentioned above are members of these pathways. For instance, the p53 pathway has been mentioned in the hubs and transcription factor analysis. The diversity and complexity of the pathways involved in these modules confirms that RA is a complex systemic disease.

### The Role of Transcription Factors

We identify 5 transcription factors in the global interaction network: FOS, FOXO1, NFAT5, NFKB1 and TP53. These transcription factors are important because not only do they link the gene regulation map with the protein-protein map as shown in Figure 2, but they also link different modules obtained from the decomposition of the map.

FOS is one of the leucine zipper proteins that can dimerize with proteins of the JUN family, thereby forming the transcription factor complex AP-1. As such, the FOS proteins have been implicated as regulators of cell proliferation, differentiation, and transformation. In some cases, expression of the FOS gene has also been associated with apoptotic cell death. Most of the genes encoding inflammatory cytokines and matrix-degrading MMPs are under the control of c-Fos/AP-1, and it plays a very important role in the destruction of arthritic joints [83]. In the decomposition of the interaction map, FOS appears in Module 2 only and MAPK14 catalyzes the activation of FOS. FOS links Modules 1, 2 and 4 and MAPK14 also links Modules 1 and 2. Neither however, are hubs in the large map. FOS is not in a closed cycle. FOS belongs to Toll-like receptor signaling pathway and the MAPK signaling pathway in Module 1 and Module 2. In Module 4, FOS is also involved in T-cell and B-cell receptor signaling pathways. The Toll-like receptor signaling pathway and MAPK signaling pathway are also very important pathways in the module pathway analysis. FOS therefore not only links the modules, but also links the important pathways of these modules. The T-cell and B-cell receptor signaling pathway also play an important role in the immune effect.

FOXO1 belongs to the forkhead family of transcription factors that are characterized by a distinct forkhead domain. It may play a role in myogenic growth and differentiation. Unphosphorylated active FOXO family proteins promote transcription of genes that regulate cell-cycle progression and survival, including CDKN1B [84], FASLG and BCL2L11 [85], which also have been listed in the map. The relationship between FOXO1 and RA has been studied in [86]–[88]. All of the aforementioned studies showed that FOXO1 contributed to the inflammation and bone destruction in the affected joints of patients with RA. Here, FOXO1 appears in the largest module, Module 4. It is connected to AKT2 and AKT2 inhibits the phosphorylation of FOXO1. AKT2 is not included in a strongly connected component in Module 4 but it has second highest degree in Module 4 and is therefore a hub. AKT2 also appears in Module 10. Neither AKT2 nor FOXO1 belong to any cycles in Module 4. FOXO1 only appears in the pathways of Module 4, it is associated with prostate cancer and insulin signaling pathway.

NFAT5 is a member of the nuclear factors of the activated T-cells family of transcription factors. This family plays a central role in inducible gene transcription during the immune response. In RA, NFAT5 mRNA is expressed in proliferating RASF but not in nonproliferating RASF. Furthermore, NFAT5 mRNA is expressed in RA synovium - but not in normal individuals - as well as at sites of bone destruction. NFAT5 could therefore be related not only with proliferation, but also with the activation and invasion of RASF *in vivo* [46]. NFAT5 links Modules 1 and 9. Despite the fact that Module 1 is relatively large (92 nodes), in the global interaction network NFAT5 has a degree of only 3, so it is not a highly connected node. In Module 9, it is linked to PTGS2 via activation of PTGS2, and PPP3CB can active NFAT5. In Module 1, NFAT5 is linked to genes FOS and JUN, which are both involved in relatively large cycles of genes (the main cycle that forms Module 1). Module 1 can be decomposed into 7 non-unique cycles, each cycle containing approximately 20 nodes. FOS appears in 4 of these cycles and JUN in the other 3. This suggests that the NFAT5 transcription pathway is closely linked to a small number of genes that have a potentially high influence on one of the largest modules, implying that perturbations made here could potentially affect a larger part of the cell. NFAT5 is not related to any pathway in the module pathway analysis.

NFKB1 is a transcription regulator that is activated by various intra- and extra-cellular stimuli such as cytokines, oxidant-free radicals, ultraviolet irradiation, and bacterial or viral products. Activated NFKB1 trans-locates into the nucleus and stimulates the expression of genes involved in a wide variety of biological functions. Inappropriate activation of NFKB1 has been associated with a number of inflammatory diseases while persistent inhibition of NFKB1 leads to inappropriate immune cell development or delayed cell growth. NFKB1 dependent gene expression in peripheral leukocytes is highly correlated with RA activity as measured by DAS28-CRP. Expression of many genes responds differentially to anti-TNF-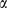 versus MTX, suggesting fundamentally different effects on the NF-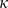
B pathway [89]. NFKB1 links Modules 1 and 2. In Module 1, NFKB1 and CCL4 work together to activate the transcription of PTGS2 in the global interaction map, which forms part of the same cycle as JUN, mentioned above. In Module 2, NFKB1 is linked to two reactions (re99:transcription and re40:heterodimer_association). The heterodimer_association of NFKBIA and NFKB1 is included in a large cycle in Module 2. In the global interaction network, genes that are directly connected to NFKB1 (inclusive) have small degree (<4) and therefore are not hubs. NFKB1 is related to the Toll-like receptor signaling pathway and the MAPK signaling pathway in Module 1. In Module 2, NFKB1 is involved in the apoptosis pathway as well as the above two pathways. NFKB1 is distributed among several pathways in Module 4 involving the MAPK signaling pathway, chronic myeloid leukemia, pancreatic cancer, the Toll-like receptor signaling pathway, acute myeloid leukemia, T-cell and B-cell receptor signaling pathway and apoptosis.

TP53 is in Module 10. It is connected to three genes: TP53 in its active state in the nucleus (through state_transition), TPPP (TPPP can phosphorylate TP53) and MDM2 (MDM2 can inhibit the activation of TP53). TP53 has a degree of 10 in both Module 10 and the global interaction network. It does not link two modules in our analysis, but is the hub of Module 10. Figure 3 shows the nodes and connections in Module 10, with the TP53 hub highlighted in red. The biological relevance of TP53 was discussed in ‘Biological Relevance of Hubs in the Molecular-Interaction Map’. TP53 only appears in the pathway of Module 10, where it is the key member of the p53 pathway.

### Analysis of Tissue-Specific Networks

The global interaction map was decomposed in to 5 sub-maps according to tissue type. The Blood_PBMC_ PMN (PBMC Peripheral Blood Mononuclear Cell, PMN Polymorphonuclear leukocytes) and the synovial_PMN sub-networks contain 3 and 16 nodes respectively. We consider these two maps to be too small for topological analysis.

**Figure 4:**
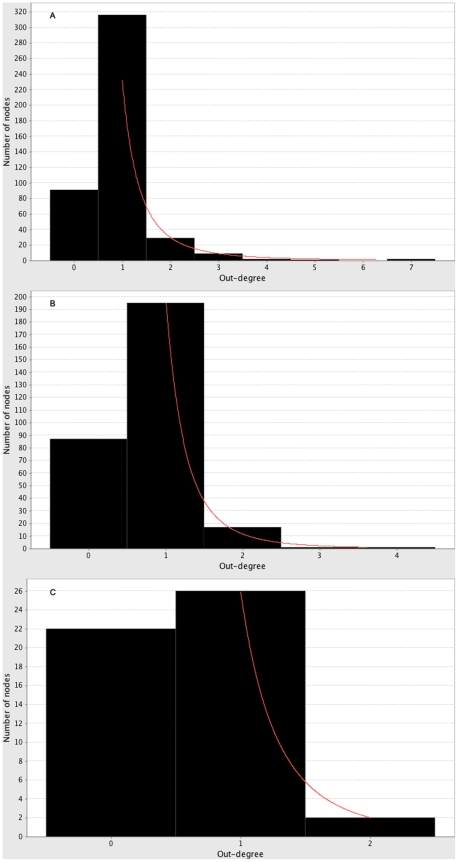
Out-degree distribution for different tissue types, fitted to power law (red line). A) Blood_PBMC (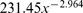), B) Synovial Fibroblast (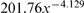), C) Cartilage (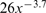). Nodes with zero degree are not included in the fitting of the power-law.

The Blood_PBMC sub-network of the RA network is the largest of the 5 tissue-specific networks, with 450 nodes and 428 edges. This sub-network is sparse, with a network density of 0.004, and an average number of 1.898 neighbours. Both the in- and out-degree distributions can be fitted to a power-law, with exponents of 2.279 and 2.964 (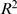: 0.972 and 0.914 respectively), suggesting the network displays scale-free properties. Figure 4A shows the out-degree distribution for Blood_PBMC map. Using degree distribution to find hubs, this network has five protein hubs (AKT2, RAC1,2, TP53, GNAI3 and CROP) with out-degree greater than 3. AKT2, RAC1,2 and TP53 are also hubs in the global interaction network (see above). GNAI3 is a hub of Module 4 as discussed above. CROP is not a drug target.

The Synovial Fibroblast sub-network contains 301 nodes and 236 edges. It is also a sparse network (though slightly less so than the Blood_PMBC sub-network), with a network density of 0.005. In- and out-degree distributions have power-law exponents of 2.279 and 4.129 (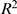: 0.97 and 0.953 respectively). Figure 4B shows the out-degree distribution for the Synovial Fibroblast map, with the power-law fitted to nodes with non-negative degree. The out-degree distribution gives rise to three protein hubs, namely IL6 and TLR2 and TLR4 (the number of hubs is reduced here due to the higher power-law exponent), with out-degree greater than 2. IL6 is discussed above and TLR2 is not a drug target. However, TLR4 is a member of the Toll-like receptor (TLR) family, which plays a fundamental role in pathogen recognition and activation of innate immunity. The TLR family recognises pathogen-associated molecular patterns (PAMPs) that are expressed on infectious agents, and mediates the production of cytokines necessary for the development of effective immunity. TLR4 is the main receptor for Lipopolysaccharide (LPS) and is most abundantly expressed in placenta, and in myelomonocytic subpopulation of the leukocytes. It has been shown that in cells expressing the 299D-399T TLR4, LPS activated the transcription factor NF
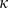
B and increased the expression of IL6 and TNF-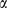. These affects were reduced by pretreatment of the cells with pravastatin or simvastatin [90].

The cartilage network is small, with 50 nodes and 30 edges. The network density is 0.024. A power-law distribution can be fitted to the in- and out-degree distributions, with exponents of 2.634 and 3.7 (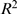: 0.987 and 1 respectively). See Figure 4C for out-degree distribution with fitted power-law. This network also has two protein hubs, IL6 and the complex containing IL12A, TNF, IL1B and IL6 (out-degree = 2). TNF is a multifunctional proinflammatory cytokine that belongs to the tumor necrosis factor (TNF) superfamily. This cytokine is mainly secreted by macrophages. It can bind to, and thus functions through, its receptors TNFRSF1A/TNFR1 and TNFRSF1B/TNFBR. TNF is involved in the regulation of a wide spectrum of biological processes including cell proliferation, differentiation, apoptosis, lipid metabolism, and coagulation. This cytokine has been implicated in RA, insulin resistance and cancers. TNF-blocking strategies are widely used in the treatment of RA. Three anti-TNF agents are registered for use in RA: etanercept, infliximab and adalimumab [91], [92]. One study has also shown that dexamethasone (DEX) contributed to the downregulation of MAM-induced IL1B and TNF-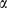 gene expression [93]. The expression of TNF was inhibited by histone deacetylase (HDAC) in affected synovial tissue [94]. In addition, the relationship between TNF and methotrexate in RA has also been studied [95]. IL1B is a member of the IL1 cytokine family. This cytokine is produced by activated macrophages as a proprotein, which is proteolytically processed to its active form by caspase 1 (CASP1/ICE). IL1B is an important mediator of the inflammatory response, and is involved in a variety of cellular activities, including cell proliferation, differentiation, and apoptosis. Glucocorticoids, such as DEX, inhibit interleukin IL1B because DEX markedly decreases the stability of IL1B mRNA. DEX was also found to be a potent inhibitor of IL1-induced expression of the IL6 gene in connective tissue-type cells from the synovium of patients with RA. Inhibition of the formation of proinflammatory cytokines, including IL1B and TNF, is a mechanism by which glucocorticoids exert anti-inflammatory effects. Inhibition by glucocorticoids of the expression of IL1B in antigen-presenting cells could decrease the capacity of the cells to stimulate the proliferation of T-lymphocytes. This activity could explain the immunosuppressive effects of glucocorticoids [66]. It has been shown that the effect of MTX treated on RA is associated with the genotype of IL1 gene. IL1RN*3 polymorphism is associated with active disease in RA patients who do not respond to methotrexate treatment, while IL1RN*long allele is associated with the worst responses to methotrexate therapy in RA patients [96]. IL12A is not a drug target.

#### Overlap Between Tissue Types

In order to be able to comment on whether there are significant topological (and hence potentially biological) differences between tissue types, we considered those nodes that appear in multiple tissue types. For all nodes that appeared in two or more tissues, we identified those nodes that had different topological properties in different tissues i.e. they are linked to different nodes in different tissues. If these nodes prove to be both topologically and biologically significant, we can draw conclusions on the necessity of targeting different tissues in the diagnosis and treatment of RA. In total, we found 58 nodes present in multiple tissue types (Table 3) - 57 in two different tissue types, and one node in three different tissue types. Of these, 29 nodes had identical nearest neighbours in both tissue types (7 isolated nodes) and 29 had different nearest neighbours in different tissue types. A biological analysis of the nodes and their corresponding pathways highlighted several important overlapping nodes as discussed below. A full list of overlapping nodes is given Table S2 and Table S3.

**Table 3:** Number of nodes shared by different tissue types.

Tissue type	B_PBMC	B+PMN	Cart	SF	S_PMN
**B_PBMC**	450	0	6	25	0
**B+PMN**	0	3	0	3	0
**Cart**	6	0	50	21	0
**SF**	25	3	21	301	4
**S_PMN**	0	0	0	4	16

B_PBMC = Blood_PBMC, B+PMN = Blood_PBMC plus PMN, Cart = Cartilage, SF = Synovial Fibroblast, S_PMN = Synovial_PMN. Some nodes were assigned to multiple tissue types. Nodes that could not be identified by tissue type were not included in this part of the analysis.

The interaction between CCNB1 and CDC2 links the Blood_PBMC map and Synovial Fibrobalst map together. CCNB1 is a regulatory protein involved in the G2/M phase of mitosis. CDC2 is a member of the Ser/Thr protein kinase family. The kinase activity of this protein is controlled by cyclin accumulation and destruction through the cell cycle. The phosphorylation and dephosphorylation of CDC2 also play important regulatory roles in cell cycle control. The complexes between CCNB1 and CDC2 form the maturation-promoting factor (MPF), which is essential for G1/S and G2/M phase transitions of eukaryotic cell cycle [97]. It has been shown *in vitro* [98] that in synovial cells, the over-expression of CCNB1 and CDC2 leads to aberrant mitosis, recognizable by abundant cytoplasm, large pale nuclei and prominent nucleoli with karyotypic alteration. These features are all typically found in synovial cells adjacent to the affected cartilage and bone of the joint in human RA and experimental animal models of arthritis. This also confirmed that rheumatoid synovial cells are ‘tumor-like’ in behaviour [99]–[101]. It should be noted that the cycle containing CCNB1 (Blood_PBMC) and CDC2 (Synovial Fibroblast) is a significant cycle both in the Blood_PBMC and Synovial Fibroblast tissues. Identification of this cycle could help us to identify the key regulatory process in the development and progression of RA.

The complex containing PB_IKK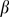, PB_IKK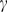 and CHUK, in the largest component of the Blood_PBMC tissue map, links to the Synovial Fibroblast tissue map. This node is a NF-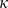
B complex, which is a key transcription factor involved in the regulation of immune responses and apoptosis. Both *in vivo* and *in vitro* studies indicate that NF-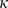
B signaling plays an important role in the development and progress of RA. Patients with RA present constitutively high serum levels of pre-inflammatory cytokines, including TNF-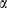, IL1 and IL6, which are NF-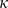
B target genes, suggesting activation of this pathway in the course of RA disease. Some activated NF-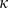
B members such as NFKB1 and RelA were found in the nuclei of cells from synovial tissue in RA patients, whereas these transcript factors were not detected in the nuclei of cells from normal synovial tissues [102]. Furthermore, the expression of NFKB1 in synovial tissue was highest at the cartilage-pannus junction, which is a site that is most likely to be associated with joint erosion [103]. Apart from the increased concentration of the active NF-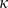
B in synovium, its binding to DNA was found to be much stronger in RA compared to osteoarthritis patients [104]. NF-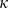
B is a potential drug target for the treatment of autoimmune diseases. Indeed, a number of novel therapeutic strategies that aim at the specific inhibition of key elements in NF-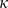
B pathway have been developed. For example, Decoy oligodeoxynucleotides (ODNs), short double stranded DNAs containing a consensus sequence for the NF-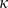
B binding sites, specifically block NF-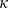
B binding to the target genes and are effective in the down-regulation of pro-inflammatory cytokine gene expression *in vitro* [105]. Apart from the specific NF-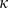
B-directed therapies, a variety of drugs used in the conventional treatment of autoimmune diseases have effects on NF-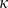
B activity. Glucocorticoiks are an example of commonly used therapeutics that can modulate the NF-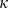
B pathway through several mechanisms mentioned in [106].

In the tissue map, TLR2 and TLR4 are shared by the Blood_PBMC tissue map and Synovial Fibroblast tissue map. In different tissue types, the various TLRs exhibit different patterns of expression. TLR2 and TLR4 are expressed most abundantly in peripheral blood leukocytes. TLRs were first suggested to have a role in RA in response to a pathogen initiating the disease [107]. Expression of TLR2 and TLR4 has been shown to be increased in the synovial tissue of RA patients compared with healthy donors or osteoarthritis samples [108], [109]. Analysis of synovial tissues of patients with RA revealed TLR2 expression in the synovial lining on fibroblasts as well as on macrophages [110]. TLR2 was found to be up-regulated by TNF-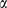 and inhibited by SB-203580 [31]. Furthermore, evidence for a TLR4 ligand in the serum and synovial fluid of RA patients has been observed. Serum and synovial fluid from RA patients stimulated TLR4 expressing CHO cells to up-regulate CD25, but the serum and synovial fluid from the healthy donors had no effect on CHO cells. In contrast, the expression of TLR4 in systemic sclerosis (SSc) and systemic lupus erythematosus (SLE) is lower than it is in RA. Furthermore, the serum from SSc and SLE patients cannot stimulate TLR4 expressing CHO cells to up-regulate CD25, which suggests that TLR4 could be a specific biomarker for RA [111]. TLR4 was also found to be associated with the response to rituximab, which is used in the treatment of many lymphomas, leukemias, and some autoimmune disorders [36]. The LY96 protein appears to be associated with TLR4 on the cell surface and confers responsiveness to lipopolysaccyaride (LPS), thus providing a link between the receptor and the LPS signaling pathway. So the interaction between LY96 in synovial tissue and TLR2 or TLR4 in the PBMC may indicate that the interaction between these two tissues in RA is mediated by the TLR signaling pathway. Synovial fibroblasts and macrophages activated by the recognition of microbial components or endogenous ligands by TLRs up-regulate the expression of proinflammatory cytokines, chemokines, and tissue destructive enzymes. In a hypothetical feedback loop, endogenous TLR ligands generated by the inflammatory processes may result in chronic stimulation of synovial cells [112].

The nodes IL1B, IL1RN, MMP1, MMP3 and TNF that overlap between synovial and cartilage tissue, as well as the nodes CCNB1, CDC2, CDK7, CCNH, IKK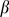, IKK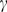 and CHUK are all involved in inflammation and immune reactions.

## Discussion

In this study we have successfully reconstructed and analysed a systemic network of interactions of the processes on-going in patients affected by RA. The network has been analysed topologically and biologically as a whole and by tissue type. The topological results show that the network is sparse, with a large number of connected components and a low number of average neighbours. Although the network follows a power-law distribution as expected, the power-law exponents are higher than is expected of a biological network (typically between 2.0 and 2.4). This means that the probability of a node having *k* connections is slightly lower than expected, resulting in fewer hubs that have a high degree. The low-density of the network, and the lower number of hubs with high degree makes the network particularly robust to change. These results, however, should be interpreted with some caution as they imply that the number of links in the network is likely to be less than the number of links that truly exist. We strongly encourage readers to add to the map where possible. The analyses should be re-run once significantly more links have been added. This, however, may take time and the map presented here can be considered as comprehensive as possible at the time of study.

In this study, we have also been interested in determining if the topologically important aspects of the network have biological significance. Hub proteins often have special biological properties: they tend to be more essential than non-hub proteins [113] and they are found to play a central role in modular organisation of the protein interaction network [114]. Furthermore, hub proteins may also be evolutionarily conserved to a larger extent than non-hubs [115]. As a result, hub proteins can be used as targets to design new drugs. Although not all topologically significant results can be explained biologically, we have found that many of the hubs identified are already drug targets for RA. This may be partly explained by the fact that these proteins and genes are well referenced and so finding the links associated with these genes is relatively easy. It is interesting to note that there do exist definite topological and biological links for the RA network, implying that the map presented here can be used to further understand how drugs influence RA at the molecular level. For example, from the topological analysis alone we can speculate that the ability to affect the rest of the network by targeting drugs at the hubs is likely to be reduced by the aforementioned topological characteristics.

One application of the map could be to determine the likely affect of targeting specific genes in the map, and to understand the effect that different drugs (that target nodes in the map) have on the rest of the network. Furthermore, those parts of the map that were topologically important, but that currently seem to have little biological relevance should be investigated further. It has been shown that drug-target proteins have higher connectivity and quicker communication with each other in a protein-protein interaction network [116], suggesting that the hubs that seem to have little biological relevance today may be potential targets for future research. Through the topological analysis of this RA network, CRKL has been identified to be a hub of Module 4. As far as searching Pharmaccogenomics Knowledge Base website ‘http://www.pharmgkb.org/index.jsp/’, we did not find that this protein has been used as any drug target. CRKL, containing SH2 and SH3 domains, had been shown to activate the RAS and JUN kinase signaling pathways. Proteins with this kind of domain-mediated protein-protein interaction are required for the transmission of proliferative signals that are initiated by tyrosine kinases. Peptidomimetic ligands based on the sequence of target proteins for SH2 and SH3 domains may represent new compounds for the therapy of proliferative diseases that are dependent upon constitutively activated tyrosine kinases [117]. Via the pathway analysis, we also found that CRKL is associated with a number of pathways, for instance, the MAPK signaling pathway, chronic myeloid leukemia and the regulation of the actin cytoskeleton pathways. Most of these pathways are related to the immune and inflammation reactions. So it is reasonable here to suggest that CRKL could be an interesting candidate as potential new drug target for the treatment of RA. This is consistent with the recommendation in [118].

Topologically speaking, it would seem that perturbations targeted at specific areas of the network will die out quickly and changes in concentrations would have to be stronger in order to reach secondary modules, if at all possible. This occurs because the average shortest path length in the interaction network is longer than expected, but as the network is not well-connected and hence the number of connected nodes is small, the power of this calculation will not be high for this network. It is also important to note that although mean path length is used as a measure in biological networks, the reader should keep in mind that as the data are scale-free, so there is skewness. Further, in the transition from *CellDesigner v4.0.1* used to draw the map, to *Cytoscape* [119], used to analyse the network (see Materials and Methods), reaction between genes and proteins are transformed from part of a link to an actual node, this means that the shortest path that can exist between a gene and a protein that interact is always 2, rather than 1 as may be seen in other interaction networks. It also means that the number of links for some nodes is reduced as they are forced to be channeled via such a reaction node. Consequently, the clustering coefficient, which can be considered in a topological analysis, cannot be calculated for the network studied here. It is beyond the scope of this project to correct this but it should be considered as an area of future research.

With the exception of the cartilage sub-map, the topological patterns seen in the interaction map as a whole can also be seen in the tissue sub-maps (see Table 1). For both the Blood_PBMC and the Synovial Fibroblast map the density is low and the power-law exponents, particularly for the out-degree distribution, are higher than is expected for a biological network. From Figure 4, it is clear that all three sub-maps have few nodes with degree higher than 2, particularly in the cartilage sub-map (Figure 4C)), which has no proteins of degree larger than 2. The low number of hubs in the graphs implies that, from the analysis of these three sub-maps, we are not able to identify many potential drug target sites within each tissue type. The higher density in the cartilage sub-map could be explained by the low number of nodes in the map. It is to be expected that Blood_PBMC has a higher number of hubs because there is more data available for blood tissue than other tissue types. Despite this, there was little topological difference between the Blood_PBMC and Synovial Fibroblast sub-maps, suggesting that there is no topological evidence to recommend that targeting one tissue type over the other is advantageous. However, the different expression levels of nodes in different tissue types might suggest otherwise. Although there is a relatively high amount of overlap between different tissue maps, only some of the overlapping nodes were shown to be biologically significant. In particular we note the existence of the CCNB1:CDC2 cycle in the Blood_PBMC and the Synovial Fibroblast tissues, which could lead to identification of the key regulatory process in the development and progression of RA if investigated further. We also note the existence of TLR2 and TLR4 in both Blood_PBMC and Synovial Fibroblast as significant as this overlap may lead us to conclude that the interaction between the two tissue types is mediated by the TLR signaling pathway.

We have successfully been able to use network analysis methods to identify biologically important areas of the network, including hubs. This supports previously published hypotheses that there is a relationship between biologically and topologically significant areas of molecular interaction maps. Without network analysis it would be impossible to visualize such important nodes or clusters in such a complex graph as the one studied here. Although the results presented here can be considered preliminary, they are, to our knowledge, the first representation of a systematic map for RA. The molecular interaction map improves our ability to understand the molecular mechanism involved in RA on the whole.

## Materials and Methods

### Data

Using the search terms ‘rheumatoid arthritis AND microarray AND expression profiling’, an intensive literature search of papers based on high-throughput RA experiments (mRNA, miRNA) was done in order to identify genes, proteins and small molecules that relate to RA. The animal studies of RA and the expression profiling performed using techniques other than microarray are deleted. Finally, 28 peer-reviewed articles had been enrolled [11]–[13], [17], [23]–[46] and combined with data publicly available in the KEGG database ‘http://www.genome.jp/kegg/’ in order to further identify genes involved in RA and to aid reconstruction of gene pathways. A total of 273 proteins, 58 genes, 46 RNAs, 5 simple molecules, 1 ion and 1 antisense RNA involved in RA were identified using this method. (See Table S4 for a tabulated summary of the literature.)

### Construction of the Molecular-Interaction Network

Using the data obtained, the general molecular interaction network was created in *CellDesigner v4.0.1* [18]. Initially, connections were built among all the molecules (proteins, genes, RNAs, simple molecules, ions and antisense RNA) presented in the literature studied. In some cases, detailed regulatory relationships between different molecules, such as activation, inhibition and phosphorylation were available, enabling the re-construction of part of the RA map. In the case where molecules were identified in the literature, but their interactions not identified (i.e. the nodes are isolated in the RA map), we searched the KEGG PATHWAY database for missing connections. Here, every molecule was input as a query term and a list of different pathways that it is involved in was obtained. For each pathway map, we were able to obtain information about the molecule's neighbours, as well as the relationships among them. If the neighbours of the queried molecule were also related to RA, then they were added to the interaction map. Otherwise, this information was not included. In different pathways, it may occur that the neighbours of the queried molecule differ. In such cases, each path is treated independently and included in the map according to the method described above. Where no interaction information was available from either the literature or KEGG database, the molecules were excluded from the map.

The resulting RA map is a directed network between molecules involved in RA, where each node represents a single molecule and links between two nodes may represent state transition (the transition of one node from one state to another state, namely activation, inhibition or phosphorylation), transcription, translation or transport. Where the transition occurs in both directions between two nodes, two directed links (one in each direction) are used. Other types of links can only occur in one direction.

### Network Decomposition and Analysis

The molecular interaction map was imported into *Cytoscape v2.6.3* for decomposition and topological analysis. Based on work previously published [120], the network was decomposed, as described below, into modules using the *Cytoscape* plugin BiNoM [121]. It should be noted here that activation or inhibition between two proteins in *Celldesigner* is represented as a node in *Cytoscape*. These nodes are given ‘re’ as a prefix, followed by an ID number assigned by the software.

As we expect the graph to have a bow tie structure [56], we first separate the map in to three sub-maps; namely a central cyclic part that contains strongly connected components (SCC), an in-component and an out-component. (All strongly connected components of the central-cyclic part were extracted using the standard Tarjan's algorithm, implemented in BiNoM.) The in- and out-components contain those parts of the map from which the central cyclic component can be reached (IN) and those that can be reached from the central cyclic component (OUT). The central cyclic part may contain several disjoint strongly connected components. Since feedback is one of the ways that an organism uses to regulate different biological networks, we decomposed the central cyclic part into relevant cycles, where a relevant cycle is defined as a cycle that is not the sum of shorter cycles [122]. Definition of relevant cycles can provide information about feedback within the network.

As each cycle is not necessarily unique, we merged those cycles that shared more than half of their nodes. This produced the core components for 11 modules that may be of biological, as well as topological, interest.

Given the core components of the 11 potentially important modules that we wish to analyse, we reintroduced the nodes (and components of nodes) that were in the IN and OUT sub-maps. When each module is merged with the IN and OUT sub-maps, only the connected parts are kept. Where components were not disjoint (i.e. were connected to multiple modules), the component was manually assigned to the module with which the majority of nodes were connected. If a component was connected to two different modules by the same number of nodes, it was assigned to the largest module. The union of all of the modules was then compared to the original graph to identify those nodes that had not been assigned to a module. This gave rise to a 12th module containing 13 connected components and 12 isolated nodes, that were disjoint from all other modules. The components in this module appear in the outer-section of the bow tie structure. The 12 modules vary greatly in size, with the number of nodes varying from 4 to 292 nodes. This implies that the original map has at least one large cluster of nodes, which is not well connected to other, smaller clusters. All modules can be downloaded in *Cytoscape* format at: ‘www.picb.ac.cn/ClinicalGenomicNTW/temp.html’.

Once the network had successfully been decomposed, we used the *Cytoscape* plugin, NetworkAnalyzer [123], to run a topological analysis on each of the 12 modules. The topological properties that we consider to be of most biological interest here are given in Table 1.

If the networks that we analyse are scale-free (i.e. they fit a power-law distribution), then their topology should be determined by a few highly connected nodes (hubs), that link the rest of the less-connected nodes to the system. Biologically speaking, nodes that can be identified as hubs in a network may lead us to be able to more readily identify important pathways in this complex network. We use both the in- and out-degree to identify hubs in the original map as well as in the larger of the 12 modules.

#### Biological Interpretation

We analysed the outputs from the topological analyses in order to determine if there was any biological significance in the topological results, in particular we use the Pharmaccogenomics Knowledge Base website ‘http://www.pharmgkb.org/index.jsp/’ and corresponding literature to determine if the nodes that were identified as being topologically important are also associated with drugs that are used to treat RA. Furthermore, we use DAVID (The Database for Annotation, Visualization and Integrated Discovery, [76]) ‘http://david.abcc.ncifcrf.gov/home.jsp’, which provides a comprehensive set of functional annotation tools for investigators to understand biological meaning behind large list of genes, to obtain KEGG pathway information of Module 1, Module 2, Module 3, Module 4, Module 6 and Module 10. The remaining five modules are too small to be able to obtain meaningful results. In the analysis, we control at the 0.01 level the number of false positives using two statistics: False Detection Rate (FDR) and Bonferroni correction (FWER).

## Supporting Information

Table S1 (0.05 MB PDF)

Table S2 (0.03 MB PDF)

Table S3 (0.03 MB PDF)

Table S4 (0.04 MB PDF)
