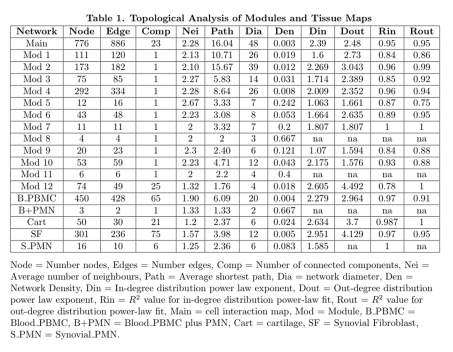# Correction: A Comprehensive Molecular Interaction Map for Rheumatoid Arthritis

**DOI:** 10.1371/annotation/f67a90fb-3e4e-4484-bffe-fcfafbfe88c7

**Published:** 2010-04-30

**Authors:** Gang Wu, Lisha Zhu, Jennifer E. Dent, Christine Nardini

A row of data is incorrect. Please view the corrected Table 1 here: 

**Figure:**