# Supplementary material for: A Comprehensive Molecular Interaction Map for Rheumatoid Arthritis
Source: PLoS One. 2010 Apr 16;5(4):e10137. doi: 10.1371/journal.pone.0010137 (PMC2855702; doi:10.1371/journal.pone.0010137)
Supplement: Table S1 — (0.05 MB PDF) [file pone.0010137.s001.pdf]

A Comprehensive Molecular Interaction Map for Rheumatoid Arthritis - S1

Table S1. Pathway Analysis of Modules 1, 2, 3, 6 and 10

Module	Pathway	Count	Total	Bonferroni	FDR
Mod 1	hsa04620:Toll-like receptor signaling pathway.	17	50	1.47E-12	9.19E-12
Mod 1	hsa04670:Leukocyte transendothelial migration.	14	50	6.78E-08	4.23E-07
Mod 1	hsa04010:MAPK signaling pathway.	18	50	3.35E-07	2.09E-06
Mod 2	hsa04620:Toll-like receptor signaling pathway.	45	64	1.58E-58	9.84E-58
Mod 2	hsa04010:MAPK signaling pathway.	27	64	8.93E-14	5.55E-13
Mod 2	hsa04210:Apoptosis.	16	64	9.83E-11	6.14E-10
Mod 2	hsa04060:Cytokine-cytokine receptor interaction.	16	64	6.61E-04	0.00412486
Mod 3	hsa04620:Toll-like receptor signaling pathway.	8	23	1.01E-04	6.29E-04
Mod 3	hsa04060:Cytokine-cytokine receptor interaction.	9	23	0.005	0.0316
Mod 6	hsa04650:Natural killer cell mediated cytotoxicity.	13	22	1.90E-11	1.18E-10
Mod 6	hsa04660:T cell receptor signaling pathway.	11	22	1.09E-09	6.79E-09
Mod 6	hsa04510:Focal adhesion.	10	22	3.52E-05	2.20E-04
Mod 10	hsa04115:p53 signaling pathway.	11	14	2.23E-14	1.44E-13

Mod = Module, Count = Number genes in pathway, Total = number of genes analysed from Module, hsa = homo sapiens, FDR = false discovery rate.
