# Supplementary material for: A Comprehensive Molecular Interaction Map for Rheumatoid Arthritis
Source: PLoS One. 2010 Apr 16;5(4):e10137. doi: 10.1371/journal.pone.0010137 (PMC2855702; doi:10.1371/journal.pone.0010137)
Supplement: Table S2 — (0.03 MB PDF) [file pone.0010137.s002.pdf]

A Comprehensive Molecular Interaction Map for Rheumatoid Arthritis - S2

Table S2. Node overlap (reaction nodes) between different tissue types

***Cytoscape* Node ID**	**Tissue 1**	**Tissue 2**
re1	syn_fibro	Blood_PBMC_PMN
re17	Blood_PBMC	cartilage
re197	Blood_PBMC	syn_fibro
re217	Blood_PBMC	syn_fibro
re258	syn_fibro	Blood_PBMC
re37	Blood_PBMC	syn_fibro
re56	cartilage	syn_fibro
re61	cartilage	syn_fibro
re66	syn_fibro	cartilage
re67	syn_PMN	syn_fibro
re70	cartilage	syn_fibro
re84	syn_fibro	cartilage
re85	syn_fibro	cartilage
re89	syn_PMN	syn_fibro

Syn_fibro = Synovial Fibroblast, Syn_PMN = Synovial_PMN, re = reaction.
