# Supplementary material for: A Comprehensive Molecular Interaction Map for Rheumatoid Arthritis
Source: PLoS One. 2010 Apr 16;5(4):e10137. doi: 10.1371/journal.pone.0010137 (PMC2855702; doi:10.1371/journal.pone.0010137)
Supplement: Table S3 — (0.03 MB PDF) [file pone.0010137.s003.pdf]

A Comprehensive Molecular Interaction Map for Rheumatoid Arthritis - S3

Table S3. Node overlap (non-reaction nodes) between different tissue types

***Cytoscape* Node ID**	**Tissue 1**	**Tissue 2**	**Tissue 3**
C_FGF11:SF_FGFR11	syn_fibro	cartilage	
C_IGF11	Blood_PBMC	cartilage	
C_IL1AA:PB_IL1R11	Blood_PBMC	syn_fibro	cartilage
C_METT:PB_VEGFCC	Blood_PBMC	cartilage	
C_NFKB1	Blood_PBMC	cartilage	
C_NFKB11:PB_MAP3K88	cartilage	Blood_PBMC	
gSF_C_IL1B@PB_nucleus	syn_fibro	cartilage	
gSF_C_IL1RN@PB_nucleus	syn_fibro	cartilage	
gSF_C_MMP1@PB_nucleus	syn_fibro	cartilage	
gSF_C_MMP3@PB_nucleus	syn_fibro	cartilage	
gSF_C_TNF@PB_nucleus	syn_fibro	cartilage	
PB_CCNB11:SF_CDC22	syn_fibro	Blood_PBMC	
PB_CCNB11:SF_CDC22 |pho	Blood_PBMC	syn_fibro	
PB_CD3EE:SF_FYNN |pho	Blood_PBMC	syn_fibro	
PB_CDK77:SF_CCNHH	syn_fibro	Blood_PBMC	
PB_F100 |active:PB_F55 |active	syn_fibro	Blood_PBMC	
PB_F100':PB_F55"	syn_fibro	Blood_PBMC	
PB_HLA-A	Blood_PBMC	syn_fibro	
PB_IKK beta	Blood_PBMC	syn_fibro	
PB_IKK betaa:PB_IKK gammaa:SF_CHUKK	syn_fibro	Blood_PBMC	
PB_IL12AA	cartilage	syn_fibro	
PB_IL1R1	Blood_PBMC	syn_fibro	
PB_ILKK:SF_PARVGG	Blood_PBMC	syn_fibro	
PB_KLRK11:PB_MICBB:SF_HCSTT	Blood_PBMC	syn_fibro	
PB_PDGFAA:SF_PDGFRAA	Blood_PBMC	syn_fibro	
PB_PIK3R55 |pho:SF_PIK3AP11	Blood_PBMC	syn_fibro	
PB_TAB11:PB_TAB22:SF_MAP3K77	Blood_PBMC	syn_fibro	
PB_TLR2,4	Blood_PBMC	syn_fibro	
PB_TLR2,44:SF_LY966	Blood_PBMC	syn_fibro	
PB_TNFRSF1A,BB:SF_C_TNFF	syn_fibro	cartilage	
rSF_C_IL1B@PB_nucleus	syn_fibro	cartilage	
rSF_C_IL1RN@PB_nucleus	cartilage	syn_fibro	
rSF_C_IL6@PB_nucleus	cartilage	syn_fibro	
rSF_C_MMP1@PB_nucleus	syn_fibro	cartilage	
rSF_C_MMP3@PB_nucleus	syn_fibro	cartilage	
rSF_C_TNF@PB_nucleus	syn_fibro	cartilage	
SF_FYN |pho |active	Blood_PBMC	syn_fibro	
SF_HCST	Blood_PBMC	syn_fibro	
SF_PDGFRA	Blood_PBMC	syn_fibro	
SPM_SF_C_IL8@default	syn_PMN	syn_fibro	
SPM_SF_PPBP@default	syn_fibro	syn_PMN	
W_SF_JUN |PB_rs1_emp	syn_fibro	Blood_PBMC_PMN	
W_SF_JUN |PB_rs1_pho |active	Blood_PBMC_PMN	syn_fibro	

syn_fibro = Synovial Fibroblast, Syn_PMN = Synovial_PMN, Blood_PBMC_PMN = Blood_PBMC plus PMN, PB_HHLA = PB_HLA-AA:PB_PDIA33:SF_B2MM@PB_endoplasmic reticulum, PB_IKK beta = PB_IKK betaa |pho:PB_IKK gammaa |pho:SF_CHUKK |pho:SF_IKBKGG, PB_IL12AA = PB_IL12AA:PB_TNF alphaa:SF_C_IL1BB:SF_C_IL66@PB_nucleus.
