# Supplementary material for: A Comprehensive Molecular Interaction Map for Rheumatoid Arthritis
Source: PLoS One. 2010 Apr 16;5(4):e10137. doi: 10.1371/journal.pone.0010137 (PMC2855702; doi:10.1371/journal.pone.0010137)
Supplement: Table S4 — (0.04 MB PDF) [file pone.0010137.s004.pdf]

A Comprehensive Molecular Interaction Map for Rheumatoid Arthritis - S4

Table S4. Literature Summary

Paper*	Tissue Type	Experiment	#
Alsaleh, '07	B_PBMC	Profiling meta-analysis between RA and SLE	5
Andreas, '08	Cartilage	Genes of RA related to destruction of cartilage	16
Antonio, '09	B+PMN	Identifying genes for rituximab response in RA	3
Arranz, '08	SF	Elucidation of the role of VIP in RA	47
Auer, '07	S_PMN	Determining whether PMN can serve as a site for CCL18 production in RA	11
Devauchelle, '04	SF	New drug targets comparing RA with OA	5
Edwards, '03	SF	Generation of a molecular description of SF comparing RA with OA	23
Galligan, '07	SF	Gene signatures involved RA and OA	4
Huang, '08	SF	Evaluation of apoptosis-associated genes in SF between RA and OA	1
Julia, '09	SF	Identifying miRNAs related to LPS-activated RA FLS	4
Junta, '08	SF	Evaluation the relationship between profiles of SF from RA patients	2
Koczan, '08	B_PBMC	Markers for etanercept treatment in RA	24
Lequerre, '06	B_PBMC	Prediction of infliximab responsiveness in RA	2
Lequerre, '06	B_PBMC	Identifying candidate gene for the early diagnosis and treatment of RA	42
Lindberg, '06	SF	Differential activation after treatment with infliximab in RA	68
Nakamura, '08	SF	Therapeutic effects of HDAC inhibitor combined with ultrasound in RA	11
Oki, '09	SF	Identification of the molecular mechanisms of bucillamine	61
Olsen, '04	B_PBMC	Signature of early RA compared to LS RA	6
Olsen, '08	B_PBMC	Gene expression profiling of PBMC of RA patients with their clinical data	3
Pohlers, '07	B_PBMC, SF	Expression profiling between RA and OA	6
Sekiguchi, '08	B+PMN	Treatment with infliximab in RA	3
Sha, '03	SF	Differential genes between RA and OA	9
Stanczyk, '08	SF	Screening miRNA in RA with TNF α	8
Teixeira, '09	B_PBMC	Identifying PBMC gene expression profiles for RA patients	18
Thierry, '09	SF	Comparison of profiles of early and LS RA	43
Timmer, '07	SF	Genes expressed in RA with ectopic lymphoid structures	11
Toonen, '08	B_PBMC, SF	Genes frequently up- or downregulated in RA	12
Zer, '07	B_PBMC, SF	Genomewide studies on the gene targets of p38 MAPK	11

*All authors are et al., # = Number Genes, B_PBMC = Blood_PBMC, S_PMN = Synovial_PMN, B+PMN = Blood_PBMC plus PMN, FLS = broblast-like synoviocytes, RA = rheumatoid arthritis, OA = osteoarthrit, LPS = Lipopolysaccharides, SLE = systemic lupus erythematosus, SF = Synovial Fibroblast, VIP = vasoactive intestinal peptide, TNF = tumor necrosis factor, HDAC = histone deacetylase, LS RA = longstanding RA, Gene = Number genes in paper.
